# COVID-19: Emergency Medicine Physician Empowered to Shape Perspectives on This Public Health Crisis

**DOI:** 10.7759/cureus.7504

**Published:** 2020-04-01

**Authors:** Christopher Gaeta, Ryan Brennessel

**Affiliations:** 1 Emergency Medicine, Children's Hospital of Philadelphia, Philadelphia, USA; 2 Emergency Medicine, Crozer-Keystone Health System, Philadelphia, USA; 3 Emergency Medicine, Bayshore Community Hospital, Holmdel, USA

**Keywords:** covid-19, public health education, physician leadership, coronavirus, novel coronavirus, severe acute respiratory syndrome coronavirus 2, community outreach, crisis management, social distancing

## Abstract

COVID-19 (Coronavirus Disease 2019) has sparked a remarkable public response in the United States. The following publication highlights the integral role that Emergency Medicine (EM) providers are afforded as a result of the public health circumstances. By embracing the unique outlet of direct patient coordination of care, EM providers can correct public misconceptions and promote more appropriate social distancing practices to the greater community.

## Editorial

COVID-19 (Coronavirus Disease 2019) has sparked a remarkable public response in the United States. As of April 1st, the United States has approximately 200,000 cases - ranking higher than any other country internationally [1]. Experts have also noted the role that social media is playing in driving false narratives about the virus [2].

Indeed, the sociocultural implications of current communications in the United States are significantly correlated with the shifting public perception of the pandemic. The bleak prospects of overcrowding emergency departments and depleting personal protective equipment (PPE) have become a reality that seemed nearly inconceivable at the onset of the outbreak in February of 2020. The seemingly growing domain of public fear presents emergency medicine (EM) physicians a rare outlet to demonstrate that patient education efforts about the virus are a vital intervention that EM physicians are uniquely equipped to spearhead.

Erroneous sets of opinions are circulating in the American public as ever-present television, social media, and radio reports are providing nearly instant updates on emerging cases, potential implications of the spread, and images showing stark shortages of hand sanitizer and N95 face masks.

**Figure 1:**
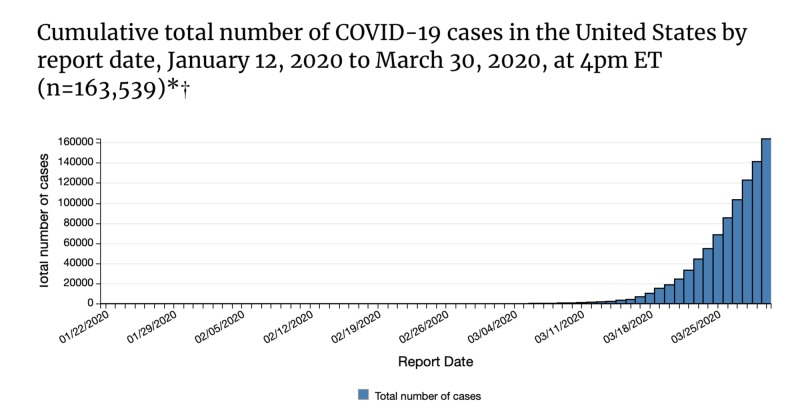
Total Number of COVID-19 Cases in the United States Between January 12, 2020 and March 30, 2020 Source: National Center for Immunization and Respiratory Diseases (NCIRD), Division of Viral Diseases

The implications of such reporting have led to a further precipitation of fears throughout the nation, a phenomenon called *argumentum ad populum*. The phrase describes the power of peer beliefs on individual perspectives. For example, the term asserts that false beliefs can be explained by the argumentum ad populum notion. This is a circumstance that explains how mass adoption of a certain perspective because of community members can be inclined to follow the behaviors of their neighbors [3]. This was particularly evident in March of 2020 as the rise of COVID-19 cases continued to increase and led to remote schooling, prohibiting mass gatherings, and temporarily suspending the operations of non-essential small businesses in many states. However, the decision to make these remarkable changes to daily life proved to save lives. Despite the telling increase in cases as shown in Figure 1, the initial efforts of mitigation should be acknowledged as a positive effort that must be further practiced as the pandemic continues to impact the nation as April approaches.

As such, communication between the masses and media outlets must involve emergency and critical care provider insight to avoid negative misinformation that could result in harmful misrepresentation cycling to the public. If individuals observe fellow community members seeking out protection from the virus by, for example, hoarding protective masks or hand sanitizer, this reality compels individuals to adopt concerns influenced by observational reactions that improperly take precedence over rational behaviors. How then, can the skewed public communication be more aligned with factual accounts coupled with pragmatic recommendations for the public to follow?

A call-to-educate patients serves as an impactful intervention that front-line emergency physicians can employ. Emergency medicine physicians must circumvent and ultimately strive to amend the misleading concerns that media outlets have the influence to potentially hinder patients with. Encouraging providers to transcend the scope of their duties is typically limited to engaging with patients to coordinate their daily care. However, authentic interactions with patients grounded in evidence-based strategies to prepare for the virus’ spread will be imperative to mitigate the inevitable overload of the healthcare system.

Emergency medicine physicians serve as a unique resource connecting a diverse patients population with emergent management of conditions. As such, they are not only called on to spearhead the initial coordination and referral of many of the COVID-19 cases but, they also have the privilege to spearhead advocacy efforts in a manner that other physicians who do not interact with this breadth of diversity in patient populations are able to match. The advocacy role of EM physicians is not limited to this public health predicament, but rather, it is a constant duty that should be further emphasized in day-to-day medical education of future EM physicians. Recent advocacy from the American College of Emergency Physicians (ACEP) represents efforts of representing EM physicians in a social and political context as the organization has successfully urged for increased PPE resources and government assistance. Patient resources compiled for COVID-19 education further depict an excellent representation of ways EM physicians can strive to educate patients, families, and peers during this pandemic [4].

Before the news of potential impacts from the virus, the operational oversight of emergency room care was struggling in general across the nation. The over-burdened emergency medical system will reach the tipping point of crisis management that will soon be in urgent need of novel delivery modalities as beds increasingly are scarce. The coming weeks will, unfortunately, echo a similar reality to that of the current effects of the virus. However, these impending increases in COVID-19 cases and economic loss might present a silver lining in the aftermath of the pandemic. Namely, reflecting on the PPE and personnel shortages experienced across the country will reinforce the overdue systematic changes that need to be addressed as we prepare for future public health crises that tax the system to the extent we are experiencing now.

Patient empowerment can best be achieved through EM physicians collaborating to promote the following three approaches to address misinformation and exaggerated media reports. Firstly, developing a well-informed, evidence-based grounding of the current, authentic information pertaining to the virus begins this process of equipping patients with facts rather than fictional convictions. Outlets to provide such updates should continue to be those that providers have relied upon historically. Reputable peer-reviewed scholarly medical journals are the large scale clinician recommendations coming from the Center for Disease Control (CDC). To garner this fundamental, updated understanding of COVID-19, medical providers can collaborate with one another on updating the community on ever-changing findings and standards of care. In doing so, they themselves are stewarding the very same lesson of seeking out credible information sources.

Additionally, as a second means of engagement, active educational outreach about the implications of the coronavirus’ foreseen spread serves as the crux of the larger call-to-action. That being, it is just as vital to equip patients with an effective, holistic care plan as it is to treat their presenting ailment in the emergency room. Having the ability to take brief moments in clinic or during cases in an emergency medicine (EM) hospital environment must be sought out to relay information on the current state of the virus. If appropriate, these brief active forms of public health stewardship will combat the damaging misinformation and subsequent harm being caused by the media’s spread of hyperbolic reporting.

Lastly, I encourage this call for public outreach to be spearheaded from emergency medicine physicians in particular. When considering the patient population alone, it is evident that EM physicians are uniquely suited to lead educational engagement because of the specialized patient population they encounter that represents a far larger proportion of their patients that are directly impacted by COVID-19 compared to many other medical specialties that are not experiencing similar volume and resource demands.

It is only when this educational outreach to any patient in the emergency department is consistently followed by providers, that a decrease in the public’s overwhelming concerns can be mitigated. The framework set out in this piece uniquely puts forth a call to action that contrasts nearly all of the other current COVID-19 investigations pertaining to best practices for clinical providers. The anticipated effects of the inevitable increase in transmission, cases, and unfortunately, fatalities in the United States can be addressed by Emergency Medicine physicians. While long-term research for vaccinations and preventative measures continue to develop, this immediate call-to-action allows EM physicians to go beyond their normal scope of care to intentionally counter the misinformation that is otherwise causing unnecessary public concerns.
